# Success and Recurrence Rate after Radial Extracorporeal Shock Wave Therapy for Plantar Fasciopathy: A Retrospective Study

**DOI:** 10.1155/2016/9415827

**Published:** 2016-07-05

**Authors:** Nikos Malliaropoulos, Georgina Crate, Maria Meke, Vasileios Korakakis, Tanja Nauck, Heinz Lohrer, Nat Padhiar

**Affiliations:** ^1^Sports and Exercise Medicine Clinic, Asklipiou 17, 54639 Thessaloniki, Greece; ^2^National Track and Field Centre, Sports Medicine Clinic of S.E.G.A.S., Thessaloniki, Greece; ^3^European Sports Care, 68 Harley Street, London W1G 7HE, UK; ^4^Centre for Sports & Exercise Medicine, Queen Mary University of London, Bancroft Road, London E1 4DG, UK; ^5^King's College London Medical School, London SE1 1UL, UK; ^6^Aspetar Orthopaedic and Sports Medicine Hospital, P.O. Box 29222, Doha, Qatar; ^7^Faculty of Sport Science and Physical Education, University of Thessaly, Karyes, 42100 Trikala, Greece; ^8^European Sportscare Network (ESN), Zentrum für Sportorthopädie, Borsigstrasse 2, 65205 Wiesbaden-Nordenstadt, Germany; ^9^Institute for Sport and Sport Sciences, Albert-Ludwigs-Universität Freiburg im Breisgau, Schwarzwaldstraße 175, 79117 Freiburg, Germany

## Abstract

*Background and Aims*. The exploration of an individualised protocol of radial extracorporeal shock wave therapy (rESWT) for plantar fasciopathy, assessing success rates and the recurrence rate over a 1-year period after treatment, is not yet identified in literature.* Methods and Results*. Between 2006 and 2013, 68 patients (78 heels) were assessed for plantar fasciopathy. An individualised rESWT treatment protocol was applied and retrospectively analysed. Heels were analysed for mean number of shock wave impulses, mean pressure, and mean frequency applied. Significant mean pain reductions were assessed through Visual Analogue Scale (VAS) after 1-month, 3-month, and 1-year follow-up. Success rates were estimated as the percentage of patients having more than 60% VAS pain decrease at each follow-up. 1-year recurrence rate was estimated. The mean VAS score before treatment at 6.9 reduced to 3.6, 1 month after the last session, and to 2.2 and 0.9, after 3 months and 1 year, respectively. Success rates were estimated at 19% (1 month), 70% (3 months), and 98% (1 year). The 1-year recurrence rate was 8%. Moderate positive Spearman's rho correlation (*r* = 0.462, *p* < 0.001) was found between pretreatment pain duration and the total number of rESWT sessions applied.* Conclusions*. Individualised rESWT protocol constitutes a suitable treatment for patients undergoing rESWT for plantar fasciitis.

## 1. Introduction

Plantar fasciopathy (PF) is a common cause of heel pain and many treatment options are available [1]. The plantar fascia is a band of connective tissue that supports the arches of the foot [2], specifically the longitudinal arch, and provides shock absorbance for the foot [3]. PF is an enthesopathy of the proximal insertion of the band [4], resulting in heel pain that is classically worse on starting activity or in the morning [3].

PF is frequently self-limiting. There are certain factors that can predispose to its development. Risk factors usually reported in the literature as leading to an increased risk of PF include high body mass index or anatomical abnormalities such as pes cavus or leg length discrepancy [5–7]. Prolonged standing and reduced ankle dorsiflexion have also been shown to influence the development of PF [8].

There are many treatment options available for PF, ranging from the conservative stretching and orthotics to the more invasive injections and, in recalcitrant cases, surgery [4]. ESWT is an alternative treatment modality that has been shown to be of benefit for PF since the 1990s [9]. Shock waves are purported to produce a controlled injury to the area resulting in neovascularisation and hence promoting healing by increasing growth factors locally [10]. Therefore, it has been proposed that ESWT is provided to patients suffering from chronic PF unresponsive to other conservative treatments [11–13]. ESWT has been shown to be beneficial for many conditions, including Achilles tendinopathy, medial tibial stress syndrome, and calcific tendinitis of the shoulder [14–16].

ESWT can be separated into two different types, radial and focused. With regard to focused extracorporeal shock wave therapy (fESWT) the waves are targeted specifically onto the affected area, whereas the waves produced by radial extracorporeal shock wave therapy (rESWT) do not concentrate on the area but instead disperse to the surrounding tissue too [17]. rESWT has been found to be of possibly more benefit than fESWT because the treatment area is larger [17], which is more beneficial for superficial injuries such as tendinopathies [18]. However there are studies that do argue for the use of fESWT over rESWT [19] or report no difference in terms of effectiveness [20]. PF is said to produce pain in a certain area rather than in a particular spot; therefore rESWT may tackle the condition better than fESWT [18].

Some studies do not provide follow-up data up to a year or more after treatment, which would help support or dismiss rESWT as a viable treatment option [18, 21]. Moreover, many studies do not specify whether rESWT or fESWT was applied [21–23]. This results in some confusion over the data. Recurrence rates for patients with PF treated with rESWT are also not well recorded in the literature. Often, there are no recurrence rates given for those who were initially treated successfully but then suffered a relapse of symptoms later on.

The aim of this study was to report the protocol used and the recurrence rate of PF after a “flexible/individualised” treatment with rESWT. The hypothesis (H_0_) that there is no relationship between pretreatment pain duration and the number of rESWT treatment sessions applied was also stated.

## 2. Methods and Materials

### 2.1. Participants

Seventy-four adults were sequentially diagnosed with PF (11 bilateral patients and 63 unilateral patients) and consented after having visited a sport and exercise medicine clinic between 2006 and 2013. All patients had a comprehensive history taken and clinical examination performed. Examination includes observation for any swelling and palpating the anatomical site of pain. This is to exclude partial plantar fascia tear (which usually occurs approximately 1.5 cm distal from the medial portion attachment), stress fracture of the calcaneus, and medial calcaneal nerve entrapment. The other useful examination is percussing (Tinel test) the inferior aspect of medial malleolus to look for tarsal tunnel syndrome [24]. Ultrasound scanning (USS) was performed in all cases in the clinic by the specialist sports medicine physician to firstly confirm the diagnosis of PF but also to review the anatomy. USS provides information on soft tissue abnormalities (e.g., synovial cysts and soft tissue induration due to fat pad contusion) but primarily allows assessment of the thickness of the plantar fascia, presence of any neovascularisation, and obvious or subtle plantar fascia tears [25].

Exclusion criteria were as follows: less than eighteen years of age, those who had undergone surgery for PF, any history of malignancy, a history of radicular back pain, any fractures in the foot, ankle, and tibia, and previous ESWT treatment.

Sixty-eight individuals (58 unilateral patients and 10 bilateral patients) were finally retrospectively analysed, 29 males (43%) and 39 females (57%) whose age ranged from 18 to 75 years with an average age of 47 ± 11 years. PF emergence differentiated in means of pain duration experienced by patients until the initialization of the treatment. We found that 21 patients (31%) experienced pain for less than three months, 14 patients (21%) experienced pain from three to six months, 18 patients (26%) experienced pain from six to 12 months, six patients experienced pain from 12 to 24 (9%) months, and nine patients (13%) experienced pain more than 24 months (Table 1).

### 2.2. Treatment Modalities

Ultrasound gel was applied to the affected area. The rESWT machine used was the Storz Medical Masterpuls MP 200 (Storz Medical, Tägerwilen, Switzerland). Application of rESWT was performed by a trained physiotherapist.

Patients were treated with an individualised protocol also dependent on their tolerance to treatment. The number of sessions, the number of impulses, the pressure, and the frequency varied between the subjects depending on the healing process and the severity and insistence of symptoms. The protocols were analysed retrospectively. However, all patients were treated for a minimum of four to six sessions. Six to eight sessions were recommended if pain existed for more than three months. One treatment was performed per week.

The working pressure was influenced by the patient's pain tolerance. If the patient was unable to cope with the set pressure due to pain, then the pressure was lowered until found acceptable by the patient. Therefore, the higher the degree of pain, the lower the pressure, although it was always set at a minimum of 1 bar.

### 2.3. Evaluation and Follow-Up

The effects of the rESWT were evaluated in all 68 patients (78 heels) over a period of one year via follow-ups arranged at 1 month, 3 months, and 1 year after treatment. Patients recorded the level of pain felt through the Visual Analogue Score (VAS) self-evaluation tool, after viewing a straight line separated in equal intervals of 1 cm, ranging from 0 to 10, where 0 represents “no pain” and 10 “worst imaginable pain” [26]. VAS scores were assessed to compute both mean VAS reductions and success rates.

**Figure 1:**
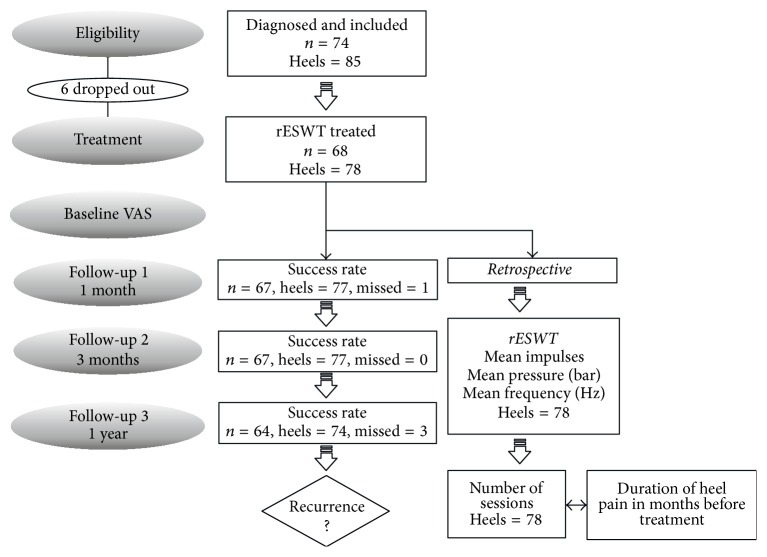
Flowchart of the research study stages. Unidirectional arrows indicate the sequential stages of the study; bidirectional arrows indicate that correlation between variables was examined.

One-year follow-up for any recurrence of symptoms was assessed during a clinic appointment. Recurrence of symptoms was defined as a painful event requiring additional cycles of treatment, or anyone with a one-year follow-up VAS score over and including four (Figure 1).

### 2.4. Statistical Analysis

The statistical analysis was performed using Microsoft Excel and SPSS 20. The frequency of the number of rESWT sessions was assessed, as well as mean shock wave impulses, mean pressure, and mean frequency applied. It was also tested as to whether the pretreatment VAS scores (baseline) were statistically significantly different from the posttreatment VAS scores with Wilcoxon Signed Ranks Test and Monte Carlo simulation to test statistical significance [27–29].

Success rates were assessed as the percentage of those having more than 60% VAS pain decrease from baseline scores [18]. Mean VAS reduction in each follow-up time interval was estimated according to the percentage of mean pain level reductions (difference in VAS scores) recorded in each follow-up time interval. In addition, mean VAS reductions were determined by dividing the difference of mean VAS pain scores by the mean VAS pain score at baseline. The percentage of VAS pain scores decrease is actually coarse metrics since they do not provide evidence about the reasons of pain healing or the significance.

Recurrence rate was assessed and Spearman's rho correlation was performed in order to test if there are grounds of approving a relationship between pain duration and the number of rESWT sessions needed. The confidence level was set at 95% (*α* = 0.05) for all statistical tests performed.

## 3. Results

From the initial number of patients included, 6 patients (5 unilateral and 1 bilateral) dropped out of the study group in the very early sessions due to financial or transportation issues, not because of the treatment itself. There were no adverse effects reported by the patients.

**Figure 2:**
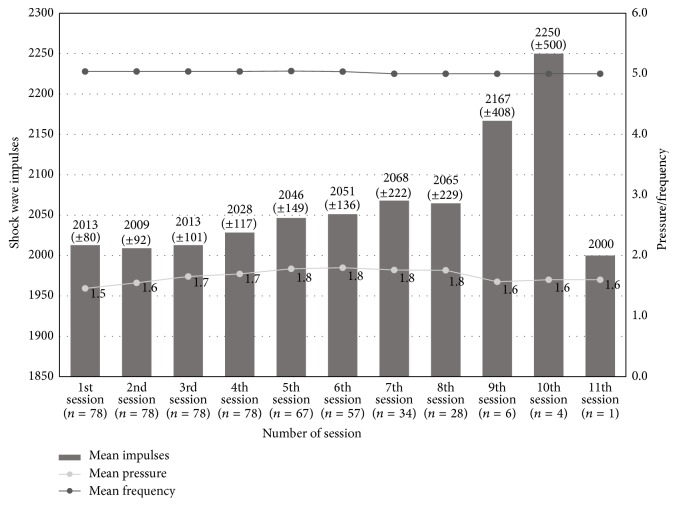
Heels rESWT mean impulses, mean pressure (bar), mean frequency (Hz), and number of patients contributing to each successive session.

The number of rESWT sessions per patient ranged from 4 to 11 with a mean of 7 ± 1.6. A total of seventy-eight (78) heels were treated with an average of 2000 impulses per session at a mean pressure of 1.7 ± 0.2 bar (ranged from 1.3 to 2.2) and a mean frequency of 5 ± 0.2 Hz (ranged from 5 to 6) (Figure 2).

From the total of 68 patients, 9 (13%) received 4 sessions, 10 (15%) received 5 sessions, 21 (31%) received 6 sessions, 5 (7%) received 7 sessions, 17 (25%) received 8 sessions, 2 (3%) received 9 sessions, 3 (4%) received 10 sessions, and 1 (1%) received 11 sessions.

There was significant reduction in VAS pain score between baseline and 1-month follow-up (*z* = −7.809, *p* = 0.000), between baseline and 3-month follow-up (*z* = −7.770, *p* = 0.000), and between baseline and 1-year follow-up (*z* = −7.615, *p* = 0.000). 3 patients did not contribute in the 1-year VAS follow-up and recurrence examination (Table 2). Negative ranks were recorded in all cases and the mean rank was found 39 at 1-month follow-up, 39 at 3-month follow-up, and 37.5 at 12-month follow-up. VAS rating was 7 at baseline, 4 at 1 month after treatment, 2 at 3 months after treatment, and 1 at 12 months after treatment.

**Figure 3:**
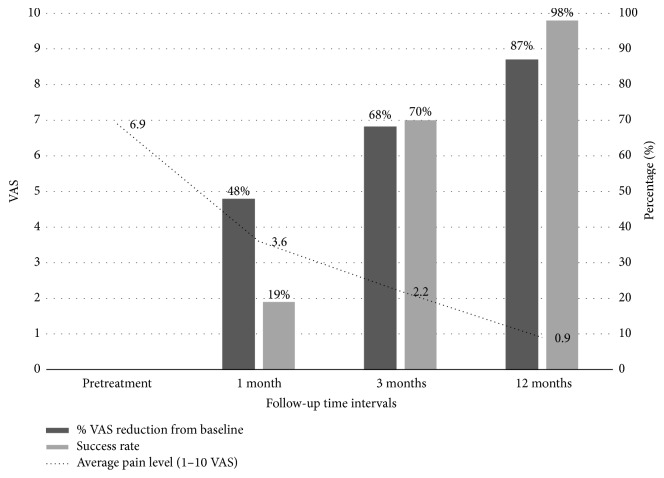
rESWT mean VAS reduction, success rate, and average pain level over 1-year follow-up intervals.

The average pain level before treatment was 6.9 and it was reduced to 3.6 one month after the last rESWT session and to 2.2 and 1.0 after 3 months and 1 year, respectively. The mean VAS reduction was 48% 1 month after treatment and 68% and 86% after 3-month and 1-year follow-up (Figure 3). Success rates were calculated at 19% (15 heels) at 1 month after treatment, at 70% (54 heels) at 3 months after treatment, and 98% (73 heels) at 1 year after treatment. The recurrence rate was 8% (5 patients out of 65) in 1-year follow-up.

Spearman's rho correlation was positive and moderate [30] between pre-rESWT treatment pain duration and the number of sessions applied (*r* = 0.462, *p* = 0.000). One year after treatment VAS was also significantly correlated with pretreatment pain duration (*r* = 0.561, *p* = 0.000) and with the number of rESWT sessions (*r* = 0.712, *p* = 0.000) performed. A positive Spearman's rho correlation was found in both cases, moderate and strong, respectively.

The Mann-Whitney *U* test was used to assess continuous variables between the group of patients with or without pain recurrence and Monte Carlo simulation to test statistical significance.

The recurrence rate was 8% accounting for 6 heels from the 74 assessed at the one-year follow-up (5 out of 65 patients). Specifically, there was one male patient with unilateral recurrence (1.4% of the feet) and four (three unilateral and one bilateral) female patients (6.8% of the feet) (Table 3).

Statistically significantly differences between the recurrence and the nonrecurrence group were found in pretreatment pain duration (*U* = 46, *p* < 0.001), in the total number of rESWT sessions applied (*U* = 97, *p* = 0.029), and in baseline VAS score (*U* = 74, *p* = 0.006).

## 4. Discussion

rESWT has already been acknowledged as an effective treatment for PF in previous studies but never in the context of an “individualised” protocol as regards the sessions applied, impulses, pressure, and frequency depending on each individual patient tolerance and response to treatment. The proposed treatment modality herein showed a 47% mean VAS reduction at 1 month, 68% at 3 months, and 86% at 1 year from the baseline, indicating good short-term and long-term results. One-year success rate at 98% revealed excellent response of patients to a modifiable rESWT treatment and the recurrence rate at 1 year was only 8%.

A serious consideration arises though when ESWT's results and its role in treating PF are compared between different published studies. A contributing factor for this is the lack of reporting as to whether fESWT or rESWT is being used. Furthermore, the current guidelines for the treatment of planar fasciitis offered by the National Institute for Health and Care Excellence (NICE, UK) include ESWT as an option, although stating that “*current evidence on its efficacy is inconsistent*” [31]. However, these guidelines were produced in 2009, and much research has been performed into ESWT since then. The guidance also does not differentiate between focused and radial [31].

When looking specifically at rESWT though, there is still some debate as to its efficacy. Whilst many studies have found rESWT highly beneficial and conclude it to be a creditable treatment option [18, 32–34], others suggest no better results than the other established therapies [19, 35–37] or not different from placebo [38]. A recent systematic review and meta-analysis concluded that “the efficacy of low-intensity ESWT is worthy of recognition” [39]. Taking the evidence into account, ESWT is effective in short- and mid-term follow-up in terms of pain and function, but its efficacy in the long term has to be established.

Possible reasons for the conflicting findings may include the different protocols used. The protocol used in this study is as follows: a thorough patient examination including ultrasonography prior to treatment; a minimum of four to eight treatments spaced one week apart depending on patients' response and the duration of the symptoms; a mean pressure of 1.7 bar, guided by the patient's pain; a mean frequency of 5 Hz; a mean total number of impulses of 2000; in our case only means and ranges are retrospectively examined. Therefore other studies which have variable numbers of treatment sessions, pressures, frequencies, and total impulses may have different outcomes to this study. Other studies have shown that multiple applications of ESWT produce better short- and long-term results than single session alone [40]. According to our protocol, the number of sessions prescribed has been shown to be enough to provoke a positive response, as seen in the results.

Summarising the differing protocols with regard to the ESWT settings, there are no standardised recommendations on the treatment parameters [34] other than those published by the manufacturers of the devices, which can vary between devices. The Storz Medical Masterpuls rESWT machine used in this study suggests the following for PF: 3–5 sessions, 2.0–3.0 bar, 3000–3500 impulses, and 12–15 Hz frequency [41]. Variations of programmes, differentiating from device instructions, have been applied in similar cases as well [19, 32, 34, 42]. These variations in protocols could therefore account for the differences found, the opinions expressed in research studies suggesting that rESWT was no better than physiotherapy [36, 37], opposite to those conveyed by others who found rESWT to be a valuable treatment modality for PF [18, 32].

In 2007 a review [43] found ESWT as a whole to be a viable treatment option for chronic PF but also commented on the varying protocols used. It found that this is the fundamental flaw with regard to ESWT research, different protocols and conflicting evidence. The contradictory findings could be the result of these different protocols, due to the large number of variables, including the populations studied, the treatment parameters, and the various outcome measures [34].

What appears to have been concluded, however, is that there has to be a balance between pressure and time: the higher the pressure the less the sessions required, but the risk of damage increases, whilst the lower the pressure the more the sessions required to see any effects [44]. Therefore it can be said that there is a dose-related relationship [44]. Therefore in this study the pressure was kept low enough to prevent any damage but high enough to have positive results. In some cases if changes were not being observed, the pressure was increased with each session until they were seen.

With regard to the 8% recurrence rate, this study found three key factors for recurrence: female sex, pretreatment pain duration, and the number of ESWT sessions received. With regard to the pretreatment pain duration, it is thought that recalcitrant PF could be caused by “plantar fascia thickening and loss of normal tissue elasticity,” that is, tissue degeneration over a period of time [45]. Therefore if a patient presents with advanced changes then they may be less receptive to conservative management. This could also explain the finding of increased recurrence with the number of sessions received (more advanced PF would require more sessions of ESWT).

Corticosteroid injections are another treatment modality recommended for PF, but there are no studies directly comparing rESWT and corticosteroid injections for the treatment of PF. Many compare injections with ESWT as a whole and have found injections more beneficial and more cost-effective [46, 47]. However, a Cochrane review concluded that whilst valuable in the short term, the effects of injection therapy are not maintained beyond six months [48]. In comparison, although expensive, some studies have concluded that ESWT has fewer complications and produces encouraging results both short- and long-term: for example, one study reported no complications and had positive short-term results (two-thirds resuming full physical activity within two months) and long-term results (6% recurrence rate within 6–12 months) [23]. Therefore at this centre rESWT is the treatment of choice.

Limitations of the present study may be found in the fact that patients were not standardised in terms of age, sex, BMI, or occupation, which could lead to analysis exploring possible correlations or depopulation. Prior to commencing the study, a sample size calculation was not performed also bringing a limitation although anticipated by a post hoc power analysis of VAS scores *t*-tests repeated measurements.

## 5. Conclusion

This study reports the recurrence rate, the mean VAS reductions, and success rates of the intervention, something not widely done in the literature. The protocol is tailored to the individual patient's needs and is hence much more flexible than other protocols used. By adapting the programme to the patient, it allows for better results as the treatment programme can progress at a rate suitable to the patient, due to the ability to allow for patient-guided feedback.

Nonetheless, this study has shown encouraging results, and therefore this rESWT protocol can be recommended for the treatment of PF. More research should be conducted with more flexible protocols such as this, in order to corroborate this study. They should also look at the implications of other variables such as gender and age, in order to assess whether programmes of rESWT can be further tailored. Apparently, meta-analysis techniques as well as the development of current good practice guidelines may assist in the fields of bringing together different outcome measures and rESWT treatment protocols on PF.

## Figures and Tables

**Table 1:** Characteristics of the patients.

Characteristic	Patients
Gender	
Male (%)	29 (42.6%)
Female (%)	39 (57.4%)

Foot affected	
Right (%)	26 (38.2%)
Left (%)	32 (47.1%)
Bilateral (%)	10 (14.7%)

Age (years) ^*∗*^mean (SD)	47.32 (±11.29)

Duration of pretreatment pain (months) ^*∗*^mean (SD)	11.21 (±59.3)

^*∗*^Values are counts (number of patients) (percentages) unless stated otherwise.

**Table 2:** VAS means, SD, and median.

	*N* (feet)	Mean	SD	Min	Max	Percentiles		
						25th	50th (median)	75th
Baseline VAS	78	6.9	0.9	5	8	6	7	8
VAS 1 month after treatment	77	3.6	1.2	2	6	3	4	4
VAS 3 months after treatment	77	2.2	1.2	0	5	1	2	3
VAS 12 months after treatment	74	0.9	1	0	4	0	1	2

**Table 3:** Patients characteristics based on recurrence.

Variable	Recurrence	*N* (feet)	Mean	SD	Min	Max	Median	Mean rank
Age	No (recurrence)	68	46.7	11.2	18	75	47	36.8
	Yes (recurrence)	6	51.0	9.3	40	60	53	45.3

Pretreatment pain duration (months)	No (recurrence)	68	10.2	10.8	1	48	6	35.2
	Yes (recurrence)	6	32.5	17.6	9	60	36	63.9

Total number of rESWT sessions	No (recurrence)	68	6.4	1.7	4	11	6	35.9
	Yes (recurrence)	6	8.0	1.4	6	10	8	55.4

Baseline VAS	No (recurrence)	68	6.8	0.9	5	8	7	35.6
	Yes (recurrence)	6	7.8	0.4	7	8	8	59.3

One-year follow-up VAS	No (recurrence)	68	0.7	0.8	0	2	1	34.5
	Yes (recurrence)	6	3.2	0.4	3	4	3	71.5
